# Citizen data sovereignty is key to wearables and wellness data reuse for the common good

**DOI:** 10.1038/s41746-024-01004-z

**Published:** 2024-02-12

**Authors:** Stephen Gilbert, Katie Baca-Motes, Giorgio Quer, Marc Wiedermann, Dirk Brockmann

**Affiliations:** 1 Else Kröner Fresenius Center for Digital Health, TUD Dresden University of Technology, Dresden, Germany; 2 The Scripps Research Institute, La Jolla, CA USA; 3 CareEvolution LLC, Ann Arbor, MI USA; 4 Robert Koch Institute, Berlin, Germany; 5 Center Synergy of Systems, TUD Dresden University of Technology, Dresden, Germany

**Keywords:** Health policy, Medical ethics

## Abstract

Smartphones, smartwatches, linked wearables, and associated wellness apps have had rapid uptake. These tools become ever ‘smarter’ in sensing intimate aspects of our surroundings and physiology over time, including activity, metabolites, electrical signals, blood pressure and oxygenation. Proposed EU law stipulates the ‘involuntary donation’ of depersonalized health and wellness data. There has been pushback against the ever-increasing gathering and sharing of wellness data in this context, increasing with every app purchased or updated. Is the potential of this data now lost to research? Consent-led COVID-19 data donation projects signpost a participative, standardized, and scalable approach to data sharing.

The 2022 legislative proposal for a European Health Data Space (EHDS) introduced bold and transformational concepts to enable citizens to take control of their health data for use in their health care^1^. It also introduced approaches for the reuse of health data for research, innovation and policy making. The most forward-thinking aspects recognized that ‘health data’ includes not only the traditional clinical data from doctor’s notes and tests but also everyday life citizen-generated data from wellness and digital health apps and wearables^2^.

## The increasing importance of ‘citizen-generated’ health data

In the last decade, many citizens have used wellness and health apps linked with their wearables which gather, interpret, and extrapolate from multi-parameter time series physiological and behavioral data. This data relates to the wellness/lifestyle domains of sleep, diet, activity, sport, and mood^3^. Data from consumer-facing domains in wellness and lifestyle are effectively inseparable from data collected from regulated health and wellness apps, interacting often with the same wearable sensors, and relating to the same physiological recordings and similar patient-generated interactions^4,5^. The EHDS proposal acknowledges three fundamental concepts that will be transformational to modern medicine, medical research and medical technology development: (i) health and data on health do not start at the clinic gate and the personal wellness category of health data describes, in intricate detail, the health of individuals; (ii) the center of gravity of health data is shifting continuously away from the clinic perimeter and towards the sofa, the pocket, the wrist and the sensor skin patch of the citizen; and (iii) if acceptable and workable approaches are found to link this data to personal EHR data, much can be learned for the understanding of diseases, developing of new approaches for early prediction and intervention in diseases and for increasing the efficiency of public health and healthcare delivery^3,6,7^.

## With great power comes great responsibility

As a result of public enthusiasm for wellness and health apps, much citizen-generated/gathered health data is already being collected. Data-collecting sensors are built into modern smart devices, and users are steered towards the activation of recording by default^8^. These data, linked to later identified diseases, can be key to the development and advancement of predictive analytics, e.g., based on AI^9–11^. Such models are applicable to rare^12^ and common diseases, either acute or chronic^3,6,7^. There is the potential for a virtuous cycle, from large-scale data sharing to large-scale provision of early and personal insights to citizens on risks, lifestyle adaptations to prevent disease, and notifications to indicate when home/clinic-based diagnostic workup is required.

The systematized and wide-scale sharing and use of citizen-generated data for developing and delivering individualized care is revolutionary but faces some understandable objections. There are fundamental questions to be addressed, regarding how data is to be gathered, used, and re-used under the EHDS proposal, and how private the private sphere of citizens will be in the future. The EHDS approach is radical in an EU context, as it proposes the sharing of patient electronic health record (EHR) data for secondary use (reuse), after depersonalization, without the basis of the consent of the citizen^2^. This would have the effect of wellness and wearables data flowing into the EHR and from there to reuse, without the explicit consent of citizens^13^. There are important concerns that should be addressed before its implementation, and, reflecting this, there has been pushback to its approach by stakeholders, including data protection supervisors. While recognizing the large potential of this data^13,14^, they emphasize that citizens must be properly informed that this data is going to be used for research. They have explicitly called for data derived from wellness applications to be excluded from all-but meaningful consent-based sharing for reuse^13^. They made this recommendation as wellness app/wearable generated data relates to every step citizens take in their everyday lives, allowing specific inferences to be made on diet, other habits, and even religious orientation^13^. In a conclusion that is likely to have a lot of influence on developing law, EU data protection supervisors call for the use and reuse of wearables data to be based solely on the prior and informed consent of the individuals, according to EU GDPR, even if the individual has uploaded this data to their own EHR^13^. Data protection supervisors are not alone in identifying this danger. Apple, a principal provider of smartphones, smartwatches, wearables, health apps, and a platform for managing the integrated data from these systems^15^, highlights a strikingly similar concern in a recent multinational media campaign on health data safety^16^. There is a risk that these challenges around data-sharing consent could lead to the most transformative aspects of the EHDS being lost. Solutions are needed that will preserve citizens’ data rights while also enabling transformative scientific discoveries.

## Learnings from COVID-19 Sovereign Data Donation

Early in the COVID-19 pandemic, wellness app and wearables data donation and reuse projects were initiated in Germany^17,18^, the UK^19^, and the US^18,20^. These projects provide important insights on how citizens engage with consent-based data donation, and also on how data donation can be practically delivered. In the DETECT study in the US^20^, citizen-controlled and app-managed consented donation of time series activity and self-reported symptoms data was shown to help identify subtle changes indicating infection^21^. The German Corona Data Donation project collected wearable data of more than 190,000 monthly active resident participants over a period of almost 3 years for detection of COVID-19 and understanding the long-term impacts of a SARS-CoV-2 infection^17^. These projects showed the high engagement of citizens for consent-based data donation when they were enabled to frictionlessly stop participation at any time and for any data type^21^. Ongoing participation tended to reduce over time^18^ and was found to be better maintained through the active engagement of the researchers with the donation community through approaches including blogs reporting project milestones or short recurring in-app surveys^22^. These projects also facilitated an international collaboration between Germany and the US in the analysis of this data^18^.

## Consented sharing for scalability and sustainability

It is not ethically acceptable or politically sustainable to harvest, without meaningful consent, more and more data from citizens, by default, with every new smart product they purchase. How can learnings from data donation projects, based on transparency, full citizen sovereignty, and self-determination over data reuse be used to build scalable and generalizable approaches for data sharing in non-pandemic crisis times? One approach would be to have numerous bespoke solutions, unique to each disease vertical, each research consortium, and to each country. This may be useful to further explore the potential of data donation, however it risks fragmentation, public confusion, and frustration of the participants. Transparent and understandable standardized solutions that enable citizens to take sovereignty over their data are needed to fulfill the promise of wellness data reuse. One proposed approach is that smartphone app-dependent wellness data collection could be linked to a trusted externally provided consent platform, allowing citizens to control and see what data they share with which destinations and for which purpose, thereby meaningfully explaining the context of health data sharing^23^. Such platforms could also be citizen engagement platforms, for reporting back to citizens and incentivizing them through information on the common good delivered through their data donation^24^. The future of health data donation from wearables is a future of fully aware and consenting participants, who understand the value of data sharing for their own good, and for the good of society, and not one of data farming from passive unaware citizens.
